# Wages of Law

**Published:** 1875-10

**Authors:** P. H. Hayes


### WAGES OF LAW.

P. H. HAYES, M. D.

I said, "Flora, you slept with your grandmother for a long time, did you not?" "Yes," said this granddaughter, still in her teens. "Well," said I, "how did it affect you?" She replied, "I felt, finally, as though my life was going out." "But what symptoms did you have?" I asked. "I don't know," said she; "I have but one impression, one memory—my life seemed going out. I left my grandmother's bed; I revived, but she died in a week's time."

A lad of about ten summers, whom I know well, was urged by his grandfather to come and sleep with him. In less than a month, this boy began to lose color, appetite and strength, and had to leave school. Being consulted, I immediately declared it my conviction, that the boy's ill-health was from sleeping with his grandfather. No sooner did this lad return to his own bed, than his appetite began to revive, and his color and strength to return, and in less than two weeks he was again in school and as well as ever.

A girl of only seven years, was permitted to sleep with her grandmother, of whom she was very fond, and as no harm to the child was ever suspected from this source, the two slept together for fourteen years. This girl belonged to a vigorous and long-lived family, and all her brothers and sisters were remarkably healthy. She, however, grew pale and puny, and died a premature death; the grandmother lived to be ninety-six years old, hale and hearty, and died of old age.

The principle here involved, that the aged and weak may draw life by contact, from the young and the strong, seems to have been well understood in Bible times, for it is written that the servants of David the King, when he was old, procured for him a youth to cherish his age, by sleeping in his bosom.

It is, however, not only true that the aged may rob the young of life, dwarf their growth, and disenchant their existence, but there are everywhere such differences in bodily health, in temperament, in vigor and elasticity of nervous system, as to make it highly probable that of any two persons sleeping habitually together, the one or the other will lose in life and spirit, and this inevitably, and the one or the other will gain in life and spirit by so much as the other loses. I believe this is true, even where both parties are, or believe themselves to be, perfectly well. I believe that much unhappiness arises in families from this source. I believe that chafings and fretfulness, among the members of many households, not to speak of partial and unaccountable and often indefinable alienations, spring from this source. I do not forget that it is quite legitimate to this reasoning, that there *might* be such a perfect adjustment, and equality of constitutional forces, between two parties, that sleeping together should, on the grounds I have named, be perfectly harmless to both, but such a state of things must be exceedingly rare, and from the nature of things, impossible to decide without an extended experience.

Every observing person will bear me out in the statement, that there is something even in the presence and society of certain individuals which to themselves is exhausting, that to spend an hour with them in close companionship is not gotten over for a whole day. I have heard this sort of complaint alike from invalids and from those in perfect health. And this, too, where the parties were dear and very loving friends. Have not every one of us in mind some person or persons, as we read these lines, who in social gatherings is one of these innocent, unconscious, but veritable mystic robbers. They, unconsciously to themselves, I suppose, rob us continually of what we were going to say; they disconcert us; we can never appear well in their presence; we are conscious of feeling weaker in some indefinable sense and manner, after they are gone, and this state of things seems quite compatible with a certain kind of admiration for their talents and character, nevertheless.

Many persons are highly emotional, exceedingly active, and impart their social life and bodily vigor so rapidly that they are perpetually in moods and tenses; they are up or down; their life is always in Elysium or Erebus. Many occupations impose upon us the duty of imparting continually. Such are those of teaching, preaching the gospel, the practice of law and medicine. These occupations are in themselves exceedingly "wearing to the nerves," and if pursued without often taking rest and turning to nature—to the fields, the streams, the woods, the mountains—for refreshment and repose, end in a more permanent exhaustion of the nervous system and a general melancholy and wretchedness.

The drift of my argument is easily understood. It is to bring out one more phase of the workings of natural law, to prove that every transgression brings with it the wages of suffering, and to lead, also, to the converse conclusion, that if we are multiplying our bodily infirmities and sufferings, it will not do in our ignorance to begin to pity ourselves, and think ourselves the subject of mysterious providences, but to make the inference at once, that we are reaping what we have sown and receiving wages.

Since writing the last paragraph, a girl of twenty years has come under my care, weak, pale, thin, and terribly dejected, and on getting her history, I find she is the youngest of a large family, and at three years of age her father died, since which time, and for about seventeen years, she has slept with her mother, between whom and herself there is a great difference in years, and, moreover, the mother has for the whole of this time been an invalid.

Such illustrations as I have given are cited to excite investigation into the often obscure causes of our bodily ill-health and our often unhappy tempers. I am fully convinced that, once attention is directed to the sleeping of any persons together, no matter what the age, sex or relationship of the parties, there will be found many and unanswerable arguments against it. I believe it is contrary to the very highest instincts of the soul and to the most perfect purity of life. I believe it has a tendency to diminish mutual respect and consideration, which are essential to exaltation of soul and a true refinement of life and manners. "A person feels elevated in proportion to the deference received from another, and there springs up a self-restraint, a consciousness of personal dignity, which has an exaltlng effect on the whole physical, moral and social nature of man." I believe the common occupation of the same bed, or the same chamber, does certainly and powerfully tend to diminish these exalting social deferences and amenities of life. As we go through our own fair land, we find in almost every family, for economy and convenience's sake, that every bed in use in the house is occupied by two persons. I believe that ill-health, secret vice, social indifference, and alienations, spring up on this account, and to a degree of which we little dream. If once the experiment could be fully made, to let every one in any family have his or her own room and bed, entirely separate from all others, I believe there would spring up a sentiment of refinement and respect, which would add immeasurably to the cheer and charm of life, and this would at once promote alike the natural health and purity of body and soul. How often, alas! how often, do we find husbands and wives have become indifferent, nay more, often fretful and disrespectful, to each other. Even where all this is outwardly restrained, we feel it; it is in the moral atmosphere of the home. Is there not always here, if the truth could be known, too much of that "familiarity," which, most of all, "breeds contempt?" Where goes the love of lovers, so often in a few months or years after marriage? Where has it flown, and why? If these questions could be patiently investigated and frankly answered, what a flood of light might be thrown on the improvement of our homes and the ways to promote the joy of our firesides!

The wages of sin—of law—is death; death *of* life *in* life.
" 'Tis *life* whereof our lives are scant;
O! *life*, not death, for which we pant—
More *life*, and fuller, that we want."

